# Indicators of Identity and Psychological Well-Being in Immigrant Population

**DOI:** 10.3389/fpsyg.2021.707101

**Published:** 2021-10-20

**Authors:** Diego Henríquez, Alfonso Urzúa, Wilson López-López

**Affiliations:** ^1^Escuela de Psicología, Universidad Católica del Norte, Antofagasta, Chile; ^2^Facultad de Psicología, Pontificia Universidad Javeriana, Bogotá, Colombia

**Keywords:** psychological well-being, group identity, ethnic identity, identity fusion, migrants

## Abstract

Multiple research has indicated that group identity processes are critical to understanding the dynamics of psychological well-being linked to migration. However, few studies have analyzed the relationship between identity from different theoretical perspectives, and the mental health of migrants in the Latin-American context. Therefore, the purpose of this study was to analyze the relationship between several identity indicators such as ethnic identity, collective self-esteem, identity fusion (with the country of origin and the host country) simultaneously, and different dimensions of psychological well-being of Colombian migrants living in Chile. The sample consisted of 887 Colombian migrants, of whom 435 (49%) were men and 452 (51%) were women. Participants were residents of the cities of Arica (*n*=204; 23%), Antofagasta (*n*=469; 52.9%), and Santiago (*n*=214; 24.1%) in Chile. The results revealed by structural equation modeling indicate that collective self-esteem and ethnic identity show positive relationships with almost all dimensions of psychological well-being, while identity fusion with Colombia only showed a positive relationship with the dimension positive relationships and identity fusion with Chile only showed a positive relationship with the dimension autonomy. Implications and limitations of these results are discussed at the end.

## Introduction

In 2020, 281 million migrants were reported worldwide [International Organization for Migration (IOM), 2020], and it is expected that by 2050 the number of people living in a country other than the one in which they were born will exceed 343 million (Chamie, 2020).

In the last decade, Chile has granted more than 1.9 million visas to foreigners [Departamento de Extranjería y Migración (DEM), 2021], which represents about 7.8% of the total population living in the country [Instituto Nacional de Estadísticas (INE) y Departamento de Extranjería y Migración (DEM), 2019]. Most migrants in Chile are from other Latin-American countries, including Colombia. Colombians are the third largest migrant population living in Chile after Peruvians and Venezuelans (Instituto Nacional de Estadísticas (INE) y Departamento de Extranjería y Migración (DEM), 2019). Despite the increase in Colombian immigration in Chile, studies that focus on inquiring about the well-being and variables related to the mental health of this population have been scarce (Urzúa and Cabieses, 2018).

The process of migrating implies a dynamic and permanent interaction between the migrant subject and the context in which he/she is inserted, which may involve various changes in both in the search for the longed-for integration in which there is a constant tension between maintaining one’s own identity or incorporating new identity elements of the host place (Arenas and Urzúa, 2016; Álvarez-Benavides, 2020). In this context, identity processes are critical to understand some psychological dynamics linked to migration, given the existing evidence of the influence of identity on people’s health and well-being (Haslam et al., 2009; Sharma and Sharma, 2010; Unger, 2011; Jetten et al., 2017; Karaś and Cieciuch, 2018).

In the present research, we address the relationship between psychological well-being and identity variables in the migrant population, such as ethnic identity, collective self-esteem, and identity fusion. These constructs become relevant once the migrant arrives in the country to which he or she migrates (Phinney et al., 2001), given their contribution to the construction of the migrant’s identity, which evolves over time. In addition, the migrant’s identity affects psychological well-being in different ways, thus altering the interaction between migrating and receiving communities (Phinney et al., 2001; Smith and Silva, 2011; Balidemaj and Small, 2019).

Well-being has traditionally been studied from two different approaches (Dodge et al., 2012). On the one hand, the hedonic approach (associated with subjective well-being) would be related to how people think and feel their existence (Deci and Ryan, 2008) through the evaluation of one’s own life through happiness (affective component) and life satisfaction (cognitive component). On the other hand, the eudaemonic approach (associated with psychological well-being) would be related to psychological fulfillment and harmony (Rodríguez-Carvajal et al., 2010), which is experienced through good living, personal growth, and development (Díaz et al., 2006). In the present study, we will deal with the psychological well-being of migrants, which refers to a network of concepts that allude to prosperity, feeling fulfilled, self-acceptance, having purposes to fulfill, and being able to make decisions autonomously (Ryff and Singer, 2008). For some authors, psychological well-being is not just a state of mind, but rather a permanent state of commitment to existence and progressive change in one’s life (e.g., Ryff, 1989; Keyes et al., 2002). In order to measure psychological well-being, Ryff (1989) proposes a multidimensional model that would reflect healthy and positive psychological functioning, which is central to understanding the psychological well-being of migrants.

In the field of migration studies on welfare and identity, ethnic identity has been one of the most studied variables (Luhtanen and Crocker, 1992; Phinney, 1992). Ethnic identity is an aspect of a person’s social identity that derives from a sense of belonging to a particular group, culture, and environment, where the actions and choices of individuals are fundamental to the process of identity formation (Phinney and Ong, 2007). Ethnic identity has been operationalized as a construct that contains two essential dimensions: exploration, which refers to efforts to learn more about one’s own ethnic group about its cultural practices by participating in them, and, on the other hand, commitment, which refers to the degree of involvement and sense of belonging that the individual maintains with his or her ethnic group, which is characterized by a high degree of personal investment in the group. Several works have presented ethnic identity as a variable that positively affects the psychological well-being of migrants (Phinney et al., 2001; Smith and Silva, 2011; Balidemaj and Small, 2019), being a protective factor against the negative effects of perceived discrimination (Brittian et al., 2015; Cobb et al., 2019; Urzúa et al., 2021) or acculturation stress (Hun et al., 2021a,b).

In general terms, there is a large body of studies that have reported the effect that ethnic identity has on people’s well-being (Smith and Silva, 2011; Balidemaj and Small, 2019). This is because identity, both individual and collective, provides people with a sense of belonging that helps maintain feelings of security, strength, competence, and self-acceptance, thus nurturing overall well-being (Haslam et al., 2009). For example, Smith and Silva, (2011) conducted a meta-analysis analyzing 184 studies on the relationship between ethnic identity and personal well-being, concluding that, despite the high variability in the magnitude of the relationship between both constructs reported in the articles reviewed, ethnic identity remained an important variable when analyzing the well-being of African-Americans, Asians, and Latinos, among others, in the US context. More recently, ethnic identity has been shown to positively relate to general well-being (Wilson and Leaper, 2016) and overall mental health (Ai et al., 2017; Turner and Llamas, 2017; Lardier, 2018). Given that ethnic identity can serve as a support base for coping with negative or stressful events (Espinosa et al., 2018), some authors have examined and evidenced that, in multicultural contexts of high proximity with other majority groups, identification with one’s own ethnic group increases the ability to emotionally connect with it, which in turn improves subjects’ emotional adjustment (Jasini et al., 2019); thus, the development of a positive identity that participates in more than one culture may help reduce negative outcomes attributable to acculturation stress. Similarly, Balidemaj and Small (2019) conducted a review of 110 articles concerning the effects of ethnic identity and acculturation on the mental health of immigrants in the USA. The authors found that overall, the studies reported that higher acculturation and ethnic identity scores were associated with higher scores on psychological well-being for migrants. Despite this, the authors indicate that there are still research gaps on the relationship between ethnic identity and well-being that remain unexplored.

Collective self-esteem is related to those aspects of identity associated with belonging to social groups and the value given to them, i.e., a valuational aspect with respect to the group (Luhtanen and Crocker, 1992). Luhtanen and Crocker (1992) propose a model where collective self-esteem is reflected in four dimensions which are: belonging or membership (i.e., how good or worthy people consider themselves as members of their social groups), private collective self-esteem (i.e., personal evaluation of how good the social groups to which one belongs are), public collective self-esteem (i.e., evaluation of how others evaluate the social groups to which one belongs), and importance to identity (i.e., how important to one’s self-concept is membership in the groups to which one belongs). Several studies have shown that collective self-esteem affects subjective (Orkibi and Bar-nir, 2015; Du et al., 2017) and psychological well-being (Crocker et al., 1994; Barrie et al., 2016) in both general and migrant populations (Verkuyten and Lay, 1998; Urzúa et al., 2019; Tartakovsky and Walsh, 2020).

Collective self-esteem and ethnic identity were born under the principles of social identity theory (Tajfel, 1982), which has been defined as the part of the individual’s self-concept that derives from the knowledge of belonging to a social group (or social groups) together with the emotional and valuational meaning associated with such belonging (Tajfel, 1984), being constructed on the basis of the perception of belonging to a group, which will be positive or negative, depending on the evaluation of the intergroup comparisons that the person makes between the in-group and the out-group (Pichastor and Nieto, 2007).

Given that both constructs have focused mainly on intergroup relations and collective ties, leaving individual relational ties that can be created with respect to the group in background (Gómez et al., 2020), we have incorporated the theory of identity fusion, a relatively emerging identity theory (Swann et al., 2009), which states that the personal self and the social self can fuse in such a way that they build relational and collective ties that emotionally and intensely bind the individual to the group (Gómez et al., 2020).

In this sense, strongly fused individuals not only value group members for the mere fact of belonging to the group, but also value group members for their idiosyncratic characteristics, going so far as to develop the perception of family ties with them, even when they do not know them personally (Swann et al., 2014a,b).

As a construct, identity fusion is associated with relational ties (feelings toward individual group members) and collective ties (feelings toward the group; see, e.g., Gómez et al., 2011a, 2020), with which a perceived connection and reciprocal strength are created between personal identity and group identity (Gómez et al., 2011a; Besta, 2018). Because of this strong unity with the group, fused individuals represent other group members as if they were their relatives (Whitehouse and Lanman, 2014; Swann et al., 2014a; Robert et al., 2019), this feeling of close bonding would be one of the main motivators of extreme pro-group behavior (see, e.g., Buhrmester and Swann, 2015; Fredman et al., 2015; Swann and Buhrmester, 2015).

Regarding its theoretical foundations, the identity fusion perspective has been based on four principles that conceptually capture its nature, namely: the agent personal self, identity synergy, relational ties, and irrevocability (Swann et al., 2012; Gómez and Vázquez, 2015); principles for which there is consistent evidence across multiple research (see, e.g., Gómez et al., 2011b; Besta et al., 2015; Vázquez et al., 2017; Heger and Gaertner, 2018).

There are few studies that have measured identity fusion in a migrant population, and those that have been found have studied the phenomenon in university students (Gómez et al., 2011a; Kiang et al., 2020); of these studies, only one evaluated fusion and its relationship with psychological well-being (Kiang et al., 2020). In contrast, other studies have evaluated identity fusion in non-migrant population associating it with variables related to psychological well-being, such as personal and social well-being, life satisfaction, and quality of life, finding, for example, that identity fusion is an important predictor of life satisfaction (Grinde et al., 2018) or that it is related to the perceived quality of life granted by the neighborhood or city (Jaśkiewicz and Besta, 2014). It has also been found that highly empowered fused individuals presented higher levels of well-being (personal and social) and community participation (Zabala et al., 2020). Finally, a cross-cultural study with more than 2,800 participants from 9 countries revealed that being fused with feminism, women or 8M (International Women’s Day) demonstrators were associated with collective effervescence, self-transcendence, and social well-being during 8M demonstrations (Zumeta et al., 2020).

Although there are studies on identity and psychological well-being in migrant populations, to date we are not aware of any research that studies identity fusion and its relationship with psychological well-being in the general migrant population. Moreover, identity fusion has been a relatively understudied construct in populations other than those from Western, educated, industrialized, wealthy, and democratic countries (Henrich et al., 2010; Henríquez et al., 2020).

Based on the above, the purpose of the present study is to examine the relationships between some identity variables (identity fusion, collective self-esteem, and ethnic identity) and the dimensions of psychological well-being (self-acceptance, positive relationships, autonomy, environmental mastery, and personal growth) in a sample of Colombian migrants in Chile. Given the existing evidence on ethnic identity, collective self-esteem, identity fusion, and psychological well-being, the following hypotheses were proposed:

Ethnic identity can act as a psychological shield (Mossakowski, 2003) and serve as a support to cope with negative or stressful events (Espinosa et al., 2018); in multiethnic environments such as those in which migrants find themselves, migrants can see their ethnic identity reinforced to create a source of personal security, social companionship, emotional ties, and association with other people of their in-group that allows them to find support for their psychological well-being and mental health (Phinney, 2003; Haslam et al., 2009). Therefore, our first hypothesis (H1) expects to find that Colombian migrants who present higher levels of ethnic identity will also present higher levels of psychological well-being in each of its dimensions (self-acceptance, H1a; positive relationships, H1b; autonomy, H1c; mastery of the environment, H1d; and personal growth, H1e).

As pointed out by social identity theory (Tajfel and Turner, 1986), identification with the group can be positive or negative according to the evaluations that the person makes of his or her social group. Therefore, for social identity to provide a sense of place, purpose, and belonging that helps maintain psychological well-being, it is necessary for the migrant to evaluate his or her group positively, that is, to have a collective self-esteem that is favorable to his or her group (Luhtanen and Crocker, 1992). It is for this reason that our second hypothesis (H2) proposes that Colombian migrants who present higher levels of collective self-esteem will present higher levels of psychological well-being in each of its dimensions (self-acceptance, H2a; positive relationships, H2b; autonomy, H2c; mastery of the environment, H2d; and personal growth, H2e).

Several studies have shown that identity fusion in different contexts offsets the possible negative effects of perceiving the group as a minority (Kiang et al., 2020; Zabala et al., 2020). Some key elements that define identity fusion are feelings of connection, reciprocal strength, agent personal self, and relational ties (Swann et al., 2009), and these elements are consistent with the idea that feeling united with a group such as country of origin (Espinosa and Tapia, 2011; Espinosa et al., 2015) would facilitate perceptions of similarity and trust by fostering reciprocal bonds of support and collective agency among members of a specific group, positively affecting people’s psychological well-being (e.g., Haslam et al., 2009, 2016, 2018). Similarly, it is likely that identity fusion by arousing a visceral feeling of group bonding could also fulfill global psychological needs (e.g., the need for belongingness or the need for meaningful existence) that are critical for maintaining healthy and positive psychological well-being functioning (Greenaway et al., 2016). Therefore, our third hypothesis (H3) posits that Colombian migrants who present higher levels of identity fusion with Colombia will present higher levels of psychological well-being in each of its dimensions (self-acceptance, H3a; positive relationships, H3b; autonomy, H3c; mastery of the environment, H3d; and personal growth, H3e).

Given that people when they arrive in a foreign country may go through acculturation processes and within those processes adopt a preferentially local identity, we decided to measure identity fusion with the host country. As demonstrated in the study Kiang et al. (2020), identity fusion with a majority group (local students) was associated with positive adaptation in a cross-cultural student context. This may be since, by being closer to the majority group, the majority group can provide resources, information, and support that could facilitate adaptation to the new school context. Following this logic, our fourth hypothesis (H4) establishes that Colombian migrants who present higher levels of identity fusion with Chile will present higher levels of psychological well-being in each of its dimensions (self-acceptance, H4a; positive relationships, H4b; autonomy, H4c; mastery of the environment, H4d; and personal growth, H4e).

## Materials and Methods

### Design and Participants

The study is correlational, non-experimental, and cross-sectional (Ato et al., 2013). A purposive sampling was carried out based on the accessibility of the participants and using the snowball strategy to recruit them. The inclusion criteria were Colombian nationality and being over 18years of age.

The sample consisted of 887 Colombian migrants living in Chile. Of these, 435 (49%) were men and 452 (51%) were women, ranging in age from 18 to 60years (*ME*=34.97; *SD*=9.59). Participants were residents of the cities of Arica (*n*=204; 23%), Antofagasta (*n*=469; 52.9%), and Santiago (*n*=214; 24.1%).

### Instruments

*Identity fusion* was measured with the verbal identity fusion scale in its Spanish version (Gómez et al., 2011a). The instrument has presented valid and reliable scores in migrant population in Chile (Henríquez et al., 2019). Because the same items were used for two different target groups, we modeled four specific factors [Feelings of Connection with Colombia (e.g., “I am one with Colombia”; 3 items), Reciprocal Strength with Colombia (e.g., “I am strong because of Colombia”; 3 items), Feelings of Connection with Chile (e.g., “I am one with Chile”; 3 items), and Reciprocal Strength with Chile (e.g., “I am strong because of Chile”; 3 items)] grouped into two general factors (identity fusion with Colombia and identity fusion with Chile). Given the similarity of the items (e.g., “I am one with Colombia” with “I am one with Chile”), the errors of the equivalent items were correlated. It was answered in a Likert response format, with options ranging from 0 (strongly disagree) to 6 (strongly agree). Higher scores reflect a higher degree of identity fusion. In the present study, the model of four specific factors (Feelings of Connection with Colombia, Reciprocal Strength with Colombia, Feelings of Connection with Chile, and Reciprocal Strength with Chile) and two general factors (Identity Fusion with Colombia and Identity Fusion with Chile) fits the data adequately (*Par*=44; *χ*2=172.176; *DF*=46; *p*=0.000; CFI=0.969; TLI=0.955; RMSEA=0.056), presenting reliable scores for all its dimensions: Feelings of Connection with Colombia=0.93; Reciprocal Strength with Colombia=0.92; Feelings of Connection with Chile=0.92; and Reciprocal Strength with Chile=0.92.

*Psychological well-being* was measured with the Ryff Psychological Well-being scale (Ryff, 1989). The adaptation of the scale was carried out by Díaz et al. (2006), a version that has already been used in Chile by researchers who have reported adequate psychometric indicators (Chitgian-Urzúa et al., 2013; Vera-Villarroel et al., 2013), including the Colombian population residing in Chile (Silva et al., 2016). However, in our study the scale presented goodness-of-fit indicators far away from those recommended by the literature (*Par*=218; *χ*2=11492.538; *DF*=362; *p*=0.000; CFI=0.673; TLI=0.633; and RMSEA=0.186; Schreiber, 2017) and anomalous correlations (*r*>1.0) in the case of the purposes dimension. It is for this reason that it was decided to debug the scale until an adequate measurement model was achieved before continuing with the structural equation model (Ruiz, 2000). Once the scale was refined, it presented goodness-of-fit indicators close to those recommended by the literature (*Par*=61; *χ*2=432.634; *DF*=109; *p*=0.000; CFI=0.920; TLI=0.900; and RMSEA=0.058) and acceptable Cronbach’s alpha coefficients (self-acceptance=0.80; positive relationships=0.70; autonomy=0.77; environmental mastery=0.63; and personal growth=0.81). Therefore, for the present study we used an *ad hoc* and reduced version of the original scale, which considered five dimensions of psychological well-being: self-acceptance (e.g., “When I look back over the history of my life, I am happy with how things have turned out”; 3 items), positive relationships (e.g., “I feel that my friendships bring me many things”; 3 items), autonomy (e.g., “I am afraid to express my opinions, even if they are contrary to what most people think”; 3 items), environmental mastery (e.g., “I have been able to build a home and a way of life to my liking”; 4 items), and personal growth (e.g., “Overall, over time I feel I continue to learn more about myself”; 3 items). The scale has a Likert-type response format of 7 options ranging from completely disagree (1) to completely agree (7).

*Collective self-esteem* was measured using the “Identity” subscale (e.g., “In general, my nationality is an important part of my image, of how I see myself”; 4 items) of the Collective Self-Esteem Scale by Luhtanen and Crocker (1992). It was answered in Likert response format, with options ranging from 1 (strongly disagree) to 7 (strongly agree). The higher the score, the greater the importance given to collective identity. In our study, the measurement model fits the data adequately (*Par*=12; *χ*2=13.693; *DF*=2; *p*=0.001; CFI=0.972; TLI=0.917; RMSEA=0.081) and the scale scores presented good reliability (*α*=0.79).

*Ethnic identity* was measured by means of an *ad hoc* scale composed of five items (e.g., “He feels very good about his cultural tradition”). These items were extracted from the Ethnic Identity scale validated by Smith (2002). In the first instance, the original scale did not present acceptable goodness-of-fit indicators (*Par*=30; *χ*2=568.276; *DF*=35; *p*=0.000; CFI=0.806; TLI=0.751; and RMSEA=0.126). Therefore, it was decided to refine the scale until acceptable goodness-of-fit indicators were obtained to perform the structural equation model. Once the scale was refined, the unifactorial measurement model was the one that presented the best goodness-of-fit indicators (*Par*=15; *χ*2=31.445; *DF*=5; *p*=0.000; CFI=0.969; TLI=0.938; and RMSEA=0.077) where the scale scores also showed good reliability (*α*=0.85). The scale was answered in a Likert-type response format, with options ranging from 1 (strongly disagree) to 4 (strongly agree). High scores reflect a strong, positive orientation toward the ethnic reference group.

*Contact* was measured by two questions, one to measure the degree of contact with other Colombians “Can you tell me how much contact you have with people from your country” and the other to measure the degree of contact with Chileans “Can you tell me how much contact you have with people from Chile.” The response options were five: (1) No contact, you only see them in the streets or in public places, but you never talk to them; (2) You see them often for neighborhood, work, or study reasons, but you do not usually talk to them, unless they address you; (3) You see them often for neighborhood, work, or study reasons, and you frequently interact with them; (4) You have friends from that group; and (5) You have relatives from that group.

### Procedure

The participants were recruited for their participation in different places of affluence of foreigners such as the Department of Foreigners and Migration, Jesuit Migrant Service, meeting places of Colombian population, agreeing voluntarily and anonymously to carry out the application after signing an informed consent. Each questionnaire was answered individually in the presence of the interviewer, to resolve any doubts regarding the understanding of the instruments. The interviewers were undergraduate thesis students, who were trained specifically for the application of the instrument. It is worth mentioning that the set of questionnaires was applied on a pilot basis prior to the study, using the cognitive interview technique, to ensure the understanding of the instruments used. The application of the battery of questionnaires lasted an average of 45min because this research was part of a larger study, which inquired about factors related to the well-being and discrimination in Latin-American immigrants in Chile. For this reason, each participant was reimbursed for their participation with an amount close to US$15 (10 thousand Chilean pesos). The instruments and the procedure were known and approved by the ethics committee of the Universidad Católica del Norte.

### Data Analysis

A structural equation model was applied estimating the effect of identity fusion (with Chile and with Colombia), ethnic identity, and identity salience on psychological well-being (self-acceptance, positive relationships, autonomy, environmental mastery, and personal growth). The analyses were carried out with the statistical software Mplus 8.2, using the maximum likelihood robust estimation method, which is robust to non-compliance with the multivariate normality assumption (Muthén and Muthén, 2017). The goodness of fit of the models was estimated using Chi-square (*χ*2) values, root-mean-square error of approximation (RMSEA), comparative fit index (CFI), and Tuker–Lewis index (TLI). According to standards recommended by the literature (e.g., Schreiber, 2017), RMSEA values≤0.08, CFI≥0.95, and TLI≥0.95 are considered adequate and indicative of good fit. Years of stay, contact with other Colombians, and contact with Chileans were controlled for in the analyses.

## Results

**Figure 1:**
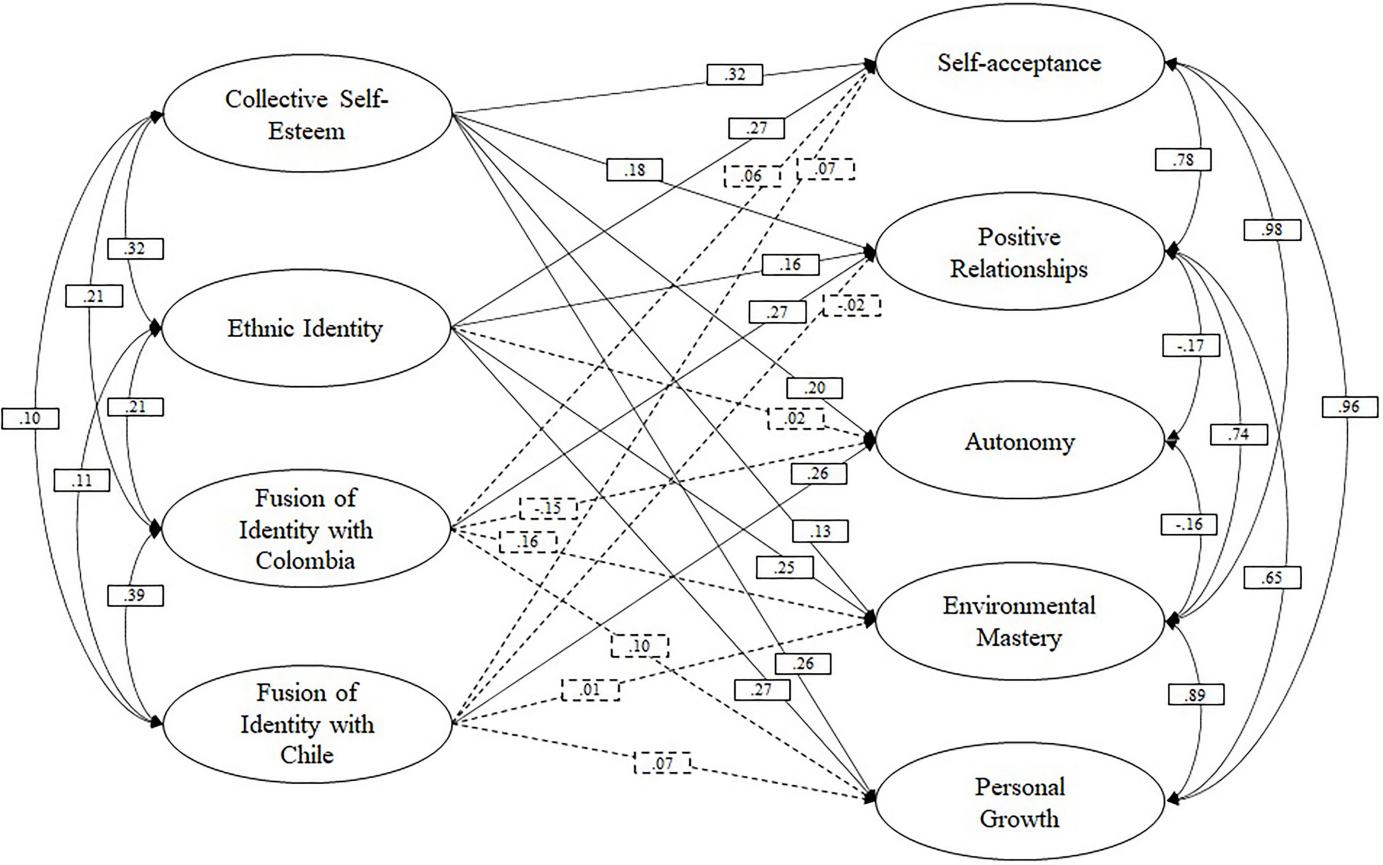
Relationship between identity constructs and dimensions of psychological well-being. The analysis controlled for the effects of years spent in Chile and the degree of contact with other Colombians and Chileans. Solid paths indicate significant relationships (*p*<0.05). Non-significant paths are shown with dashed line.

Table 1 shows some descriptive statistics (*n*, *ME*, *SD*) and the correlations of all the variables included in the model. The hypothesized model presented goodness-of-fit indices close to the criteria recommended by the literature (*Par*=181; *χ*2=2186.823; *DF*=721; *p*=0.000; CFI=0.910; TLI=0.897; and RMSEA=0.048). Therefore, the proposed model is a good representation of the observed relationships (Figure 1).

**Table 1:** Scores and the correlations of the variables included in the model.

Variables	*n*	*ME*	*SD*	CSE	EI	SA	PR	AU	EM	PG	FC_COL_	RS_COL_	FC_CHI_
Collective Self-Esteem	875	5.36	1.22										
Ethnic identity	870	3.44	0.59	0.28^*^									
Psychological Well-Being													
Self-acceptance	866	5.32	1.21	0.36^*^	0.33^*^								
Positive relationships	856	4.84	1.31	0.22^*^	0.21^*^	0.61^*^							
Autonomy	874	3.80	1.49	−0.10^*^	−0.05	−0.10^*^	0.07^*^						
Environmental mastery	871	5.11	1.29	0.18^*^	0.26^*^	0.69^*^	0.51^*^	0.07^*^					
Personal Growth	877	5.45	1.35	0.30^*^	0.32^*^	0.77^*^	0.52^*^	−0.08^*^	0.68^*^				
Identity fusion													
Feelings of connection with Colombia	854	3.66	1.67	0.21^*^	0.23^*^	0.19^*^	0.27^*^	−0.03	0.17^*^	0.20^*^			
Reciprocal strength with Colombia	856	3.45	1.73	0.16^*^	0.12^*^	0.17^*^	0.26^*^	0.05	0.21^*^	0.18^*^	0.80^*^		
Feelings of connection with Chile	842	2.24	1.53	0.04	0.13^*^	0.12^*^	0.09^*^	−0.21^*^	0.05	0.11^*^	0.38^*^	0.23^*^	
Reciprocal strength with Chile	841	2.17	1.52	0.03	0.08^*^	0.13^*^	0.07^*^	−0.13^*^	0.11^*^	0.14^*^	0.31^*^	0.42^*^	0.78^*^

* *p*<0.05;

CSE=Collective Self-Esteem; EI=Ethnic identity; SA=Self-acceptance; PR=Positive relationships; AU=Autonomy; EM=Environmental mastery; PG=Personal Growth; FC_COL_=Feelings of connection with Colombia; RS_COL_=Reciprocal strength with Colombia; and FC_CHI_=Feelings of connection with Chile.

In the case of ethnic identity, it has significant and positive relationships with four of the five dimensions of psychological well-being. The magnitudes of these correlations ranged from slight [positive relationships (H1b)] to small [self-acceptance (H1a), environmental mastery (H1d), and personal growth (H1e)]. Ethnic identity did not show significant relationships with autonomy. These results partially support the first hypothesis, since only H1a, H1b, H1d, and H1e were fulfilled.

Specifically, it can be observed that collective self-esteem has significant and positive relationships with all dimensions of psychological well-being. The magnitudes of these correlations were slight [*b*>0.10, Cohen, 1988; positive relationships (H2b) and environmental mastery (H2d)], small [*b*>0.20; Cohen, 1998; autonomy (H2c) and personal growth (H2e)], and moderate [*b*>0.30; Cohen, 1988; self-acceptance (H2a)]. Therefore, these results provide full support for the second hypothesis (H2a, H2b, H2c, H2d, and H2e).

Finally, in terms of identity fusion, the fusion with Colombia only had a statistically significant, positive, and small relationship with the dimension positive relationships (H3b), while the fusion with Chile only had a statistically significant, positive, and small relationship with the dimension autonomy (H4c). The third and fourth hypotheses had little support, being observed only in H3b and H4c.

## Discussion

Given the prominent migratory boom that Chile has experienced in recent years and the scarce studies related to the mental health of migrants who have settled in Chile, it is necessary to have studies on the subject (Urzúa and Cabieses, 2018). The purpose of the present study was to examine the relationship between collective self-esteem, ethnic identity, and identity fusion (with Colombia and with Chile) and dimensions of psychological well-being (self-acceptance, positive relationships, autonomy, environmental mastery, and personal growth) in Colombian immigrants residing in northern and central Chile. For a better understanding of migrants’ identity, in the present study we used two theoretically different identity perspectives (Gómez et al., 2020), the social identity theory (Tajfel, 1972) and the identity fusion theory (Swann et al., 2009). The results indicate that multiple facets of migrants’ identity (collective self-esteem, ethnic identity, and identity fusion with Colombia and Chile) have differentiated but positive relationships with their psychological well-being and its component dimensions (self-acceptance, positive relationships, autonomy, mastery of the environment, and personal growth).

First, it was observed that ethnic identity presented positive correlations with positive relationships, self-acceptance, mastery of the environment, and personal growth, partially fulfilling the first hypothesis. Therefore, a high level of ethnic identity can positively influence the psychological well-being of migrants. This can be explained because ethnic identity becomes relevant in multiethnic environments and where individuals perceive their ethnic groups as minorities vis-à-vis the dominant ethnic culture (Balidemaj and Small, 2019). In addition, ethnic identity could be acting as a psychological shield (Mossakowski, 2003) and serve as a support to cope with negative or stressful events (Espinosa et al., 2018) such as exclusion and discrimination (Urzúa et al., 2019). Other authors posit that ethnic identity promotes social companionship, emotional bonds, and association with others of the same ethnic group, allowing finding peer support to maintain healthy mental health (Phinney, 2003; Haslam et al., 2009). In addition, Colombian migrants in Chile often share common experiences due to the need for adaptation and the existence of cultural and social status contrasts with the dominant culture. For this reason, many times migrants can be objects of ethnic and/or racial discrimination and can collectively fall into a network of social disadvantages (Urzúa et al., 2019). In this way, migrants perceive that the social strata, living conditions, and historical experiences are like that of other Colombian migrants. These feelings could create a greater identification with other Colombian migrants, which would help to maintain part of their self-acceptance through positive relationships, thus facilitating, in this way, their personal growth through a better mastery of the environment. It is worth mentioning that no evidence was found that ethnic identity was related to autonomy, perhaps due to the precarious conditions that Chile provides to migrants arriving in the country, so that migrants are often subject to co-dependence and mutual help among people of the same ethnic group within the country, explaining the greater weight of collaborative relationships over autonomy in the well-being of the migrant population.

Second, positive correlations were observed between collective self-esteem and self-acceptance, positive relationships, autonomy, mastery of the environment, and personal growth of Colombian migrants residing in Chile. Therefore, our second hypothesis was fully supported. From the theory of social identity (Tajfel and Turner, 1986), it is suggested that identification with one’s own group can be positively or negatively valued. Because of this mechanism, for social identity to provide a sense of place, purpose, and belonging that helps maintain psychological well-being, it is necessary for the migrant to evaluate his or her group positively, that is, to have a collective self-esteem that is favorable to his or her group (Luhtanen and Crocker, 1992). It is for this reason that there is a strong relationship between identification with one’s own group and collective self-esteem. In this sense, migrants strive to maintain a positive social identity in order to increase their personal and collective self-esteem (Phinney et al., 2001). Thus, there are studies that indicate that collective self-esteem is a mediator of the relationship between perceived social status and psychological well-being (Verkuyten and Lay, 1998), or that it is a buffer against perceived ethnic discrimination (Kong, 2016). Studies in the Chilean context have indicated that both individual and collective self-esteem of migrants partially reduces the effects of discrimination on well-being (Urzúa et al., 2018) and anxious and depressive symptomatology (Urzúa et al., 2019) of the migrant population. Thus, we can think that collective self-esteem functions as a protective resource in which ethnic minorities amplify the positive characteristics of their group in order to feel good about themselves and the collective, they feel part of, which would translate into an increase in their overall psychological well-being.

Third, identity fusion with Colombia was positively related to positive relationships. Although this was one of the most exploratory hypotheses due to the scarce literature on the subject, these results are relevant because they are the first evidence that identity fusion with the country of origin (in the case of migrants) is only related to one of the dimensions of psychological well-being (positive relationships). This may suggest that migrants who maintain or build strong relational and collective ties with other Colombians are better able to maintain warm, satisfying and trusting interpersonal relationships even while in Chile. The mechanisms that promote this relationship are likely to be due to perceptions of similarity and trust that foster supportive reciprocal ties and personal or collective agency among Colombian migrants living in Chile, positively affecting individuals’ psychological well-being (e.g.,; Haslam et al., 2009), given that it is very likely that the fusion could be fulfilling psychological needs such as the need to belong or the need for a meaningful existence that are fundamental to maintaining healthy and positive psychological functioning (Greenaway et al., 2016).

Finally, as for identity fusion with Chile, this was positively related only to autonomy, suggesting that migrants who feel deeply attached to Chile also have an easier time developing in an independent and self-determined manner within the national context. As demonstrated in the study Kiang et al. (2020), identity fusion with a majority group was associated with positive adaptation in an intercultural student context. This may be since by being closer to the majority group, the majority group can provide resources, information, and support that could facilitate adaptation to the new context.

It is important to pay attention that both the fusion with Colombia and the fusion with Chile do not reflect positive or negative relationships on the rest of the dimensions of psychological well-being of the migrants (self-acceptance, mastery of the environment, and personal growth). It would be important to investigate the existence of possible mediating or moderating variables that could create the connection between identity fusion and the totality of the dimensions of psychological well-being presented by migrants. Moreover, the importance of these results lies in the fact that studies on identity fusion and well-being in general are scarce. To the best of our knowledge, we know of only one paper that analyzed the relationship between identity fusion and psychological well-being (Kiang et al., 2020). The differences between our study and that of Kiang et al. (2020), consisted of in that: our study was based on data from a non-university migrant sample, whereas Kiang et al. (2020) analyzed data from a sample of international students. In our study, we analyzed the relationship between identity fusion and specific dimensions of psychological well-being by modeling it as latent variables rather than observable variables (Kiang et al., 2020). On the other hand, to measure identity fusion, we used the verbal measure, whereas Kiang et al. (2020) used the pictorial measure. Finally, we relied on extended fusion, using home and host country as reference groups, whereas Kiang et al. (2020) relied on local fusion, using domestic students (or local students) and international students as reference groups. However, despite these differences, the results of both studies supported the idea that identity fusion has benefits for the psychological well-being of the participants.

In conclusion, ethnic identity, collective self-esteem, and identity fusion are identity indicators that manage to explain, from different theoretical perspectives, the self-acceptance, positive relationships, autonomy, mastery of the environment, and personal growth of Colombian migrants in Chile. In agreement with previous findings, variables such as ethnic identity and collective self-esteem, which are based on classical social identity theory, show a greater number of relationships with the dimensions of psychological well-being of migrants. On the other hand, identity fusion presented a low number of relationships with the dimensions of psychological well-being. Like the results presented by Kiang et al. (2020), it may be that fusion is an important factor to consider in areas related to well-being; however, its influence may not be broad enough to explain all dimensions of psychological well-being.

## Limitations

Although the present study may be of great relevance, it is important to point out some limitations of these results. First, it should be considered that the study was cross-sectional in nature, so that the relationships between variables cannot be attributed causal effects. Second, the sample was biased toward Colombian migrants who wanted to participate in the study, so the conclusions that could be reached would only be plausible for a population like that of the sample and could not be generalized to migrants from other countries. Finally, it is important to keep in mind that although the scales used in the present study presented measurement models with good fit indices, some of these scales (ethnic identity, collective self-esteem, and psychological well-being) are short *ad hoc* versions for this study. Therefore, it would be important to have further studies that analyze the psychometric properties of the short versions presented in this study.

## Data Availability Statement

The raw data supporting the conclusions of this article will be made available by the authors, without undue reservation.

## Ethics Statement

The studies involving human participants were reviewed and approved by Comité Ético Científico of Universidad Católica del Norte. The patients/participants provided their written informed consent to participate in this study.

## Author Contributions

DH and AU contributed to conception and design of the study. DH organized the database, performed the statistical analysis, and wrote the first draft of the manuscript. DH, AU, and WL-L wrote sections of the manuscript. All authors contributed to manuscript revision, read, and approved the submitted version.

## Funding

This publication is derived from the project FONDECYT Regular #1180315, funded by the Agencia Nacional de Investigación y Desarrollo (ANID) of the Government of Chile.

## Conflict of Interest

The authors declare that the research was conducted in the absence of any commercial or financial relationships that could be construed as a potential conflict of interest.

## Publisher’s Note

All claims expressed in this article are solely those of the authors and do not necessarily represent those of their affiliated organizations, or those of the publisher, the editors and the reviewers. Any product that may be evaluated in this article, or claim that may be made by its manufacturer, is not guaranteed or endorsed by the publisher.
